# A Case of Poisoning by Corrosive Sublimate

**Published:** 1850-07

**Authors:** 


### ECLECTIC DEPARTMENT,

### AND SPIRIT OF THE MEDICAL PERIODICAL PRESS.

---

*A Case of Poisoning by Corrosive Sublimate.* By BENJ. W. MCCREADY, M. D., of New York. Communicated by Dr. T. R. BECK.

I was called on Friday, December 28, about 11 P. M., by my friend, Dr. G. O. Gunn, to see with him a young woman who had poisoned herself with corrosive sublimate. According to the account of the family, she had taken tea with her father and brother as usual, between six and seven o'clock in the evening, and immediately afterward had retired to her own room. About ten P. M., her brother going up stairs, had his attention attracted by the noise she made in her room, and on entering it, he found her vomiting and retching violently. On being questioned, she stated, that immediately after tea she had poured out a cup full of an alcoholic solution of corrosive sublimate, which was kept in the room as a bug poison, and had swallowed the whole of it. The solution was of the strength of a drachm of the salt to half a pint of alcohol, and as she had swallowed about three ounces of it, she had consequently taken about 22½ grains of the poison. Dr. George O. Gunn was immediately sent for. The vomiting was encouraged by draughts of warm water, and a bowl full of white of eggs was prepared and administered, though from the length of time that had elapsed since the poison had been taken, and the copious vomiting that had since occurred, Dr. G. did not hope for much good effect from their employment.

I found the patient exceedingly restless, with a cool skin, an anxious countenance, and a frequent, feeble pulse. She made no complaint, but when questioned, said that she had a burning pain in her stomach, and that she found a good deal of difficulty in swallowing. There was great thirst, with retching and vomiting. The tongue was pale, but there were some bright red spots, with a well defined margin in the posterior fauces. A full opiate was ordered, with ice and diluent drinks, together with hot fomentations to the abdomen.

On Saturday, to our surprise, Dr. G. and myself found her with a pale but tranquil countenance, the pulse about ninety, and of good volume, the skin cool and the thirst abated. She had two stools during the night, feculent, but containing a little blood. Simple diluents were continued, and it was agreed to administer opiates according to circumstances.

From this time (Saturday,) I did not see the patient again until the following Friday, January 4th. According to Dr. Gunn, she had slight dysentery during Sunday and Monday, having each day five or six mucous passages with a little blood. From this time she had but one stool a day. On Monday suppression of urine occurred, and from Monday till Friday the patient passed no urine at all. The catheter then having been introduced, Dr. G. drew off less than four ounces of somewhat turbid urine. Her skin remained, during the whole time, cool, and the pulse was never above ninety. During Monday, Tuesday and Wednesday the patient did not vomit, but on Wednesday evening she ate, through the injudicious indulgence of an attendant, two tea buiscuit, and from this time vomiting recurred, consisting chiefly of the fluids drunk, mingled occasionally with a little dark grumous matter. The patient made no complaint, but appeared to become weaker. She slept a good deal, but was easily roused, and occasionally seemed to wander slightly in her mind. Her gums had become somewhat swollen, and there was slight salivation. I found her with a dull and listless expression of countenance and a feeble pulse, about 90 in a minute. She made no complaint on firm pressure being made over the abdomen. There was frequent vomiting and great prostration of strength. On the following day she died.

A post mortem examination was made twenty-four hours after death by Dr. Holmes, the assistant of the coroner. The abdomen alone was opened, and even of this the examination was very imperfect. Externally the stomach, the small and large intestines appeared very miuutely injected with blood. The bladder was contracted and contained no urine. I obtained from Dr. H. the stomach, a portion of the ascending colon, and the kinneys. The stomach was much injected, the mucous membrane softened, and in spots slightly eroded. The portion of the colon was vividly injected, the mucous membrane thickened, and between the longitudinal bands there were a number of well defined rounded ulcerations with elevated edges, about the third of an inch in diameter, and extending completely through the mucous membrane. One kidney was much enlarged, and presented evidences of slight congestion. On the surface of the other kidney, several serous cysts had been developed, causing atrophy of its proper substance. One of these cysts had burst, and had separated extensively its capsule from the kidney; turbid serum with a white curdy matter escaping when the capsule was cut into. The other cysts on being opened were found to be filled with a similar matter.

The case is note-worthy on several accounts.

1st, The general symptoms bore no relation to the amount of local disease. After the first few hours, the pulse was moderately full, and not above ninety, and the temperature not unusual. This might be connected with the state of the blood after the occurrence of the suppression of urine, but it was present previous to the coming on of the suppression.

2d, The amount of inflammation and ulceration of the large intestines was such as occurs commonly with severe dysentery, and yet the dysenteric symptoms were slight, much slighter, indeed, than commonly occur in cases of poisoning by corrosive sublimate.

3d, Suppression of urine continued from Monday until Saturday, the day of her death, and yet the patient retained her senses, and was easily roused, exhibiting no signs of cerebral disturbance except slight drowsiness, and some tendency to wandering.—*Transactions N. Y. State Med. Society.*

